# Stop talking about it already! Co-ruminating and social media focused on COVID-19 was associated with heightened state anxiety, depressive symptoms, and perceived changes in health anxiety during Spring 2020

**DOI:** 10.1186/s40359-022-00734-7

**Published:** 2022-02-07

**Authors:** Lindsey B. Stone, Alice E. Veksler

**Affiliations:** 1 Department of Psychology, Georgia Southern University, PO Box 8041, Statesboro, GA 30460 USA; 2 Department of Communication, Christopher Newport University, Newport News, USA

**Keywords:** COVID-19, Mental health, Co-rumination, Social media, Health anxiety

## Abstract

**Background:**

Social distancing presents a significant obstacle for relationships and threatens mental health. Identifying maladaptive, voluntary coping strategies may inform how to maintain interpersonal relationships and mental health during quarantine. Co-ruminating with peers on negative events, moods and fears has adjustment trade-offs of increasing depression and anxiety risk while also enhancing friendship quality. Similarly, social media use is associated with social benefits and risk to mental health. We extend prior research by examining whether co-ruminating on COVID-19, social media use, and social media use focused on COVID-19 during social isolation was associated with heightened depression and anxiety symptoms but also lower loneliness and higher social support during initial lockdown measures in the USA.

**Methods:**

Adults were recruited through social media (*n* = 345) to complete self-report surveys on co-rumination, social media use, social distancing, social support from March–May 2020. During this cross-sectional assessment, in addition to completing surveys on current depressive symptoms and state and health anxiety, participants also provided retrospective report of their perceived health anxiety levels six months prior.

**Results:**

Co-ruminating on COVID-19 with peers and greater time on social media focused on COVID-19 predicted perceived increases in health anxiety and were also associated with higher depressive symptoms and state anxiety, even after controlling for significant demographic predictors. Further, in the context of social distancing, both interaction strategies failed to confer social benefits.

**Conclusions:**

Results have direct implications for maintaining psychosocial health during social distancing restrictions. Adults may modify how they engage with peers by limiting COVID-19 content on social media and COVID-19 discussion.

## Background

In 2019, a novel and highly infectious strain of the coronavirus, SARS-CoV-2, was identified in the Wuhan province of China. Within months, COVID-19, proliferated across the globe leading to the World Health Organization declaring a global pandemic in March of 2020 [1]. For most, this pandemic presents the largest public health threat in our lifetime. To date, even with the recent emergency use authorizations of COVID-19 vaccines for use in the US [2], the primary defense against COVID-19 has been (and remains to be) masking and social distancing which is defined as staying 6 feet apart from other people, not gathering in groups, staying out of crowded places and avoiding mass gatherings [3]. The directive to social distance resulted in ‘stay at home’ and ‘shelter in place’ orders across the world. Within the US, Governors shuttered businesses and limited access to public spaces, significantly curtailing in-person interactions, which poses significant risks for mental health. Given that not all social interaction strategies are adaptive, we sought to identify whether volitional (and hence modifiable) interaction strategies for maintaining social relationships during quarantine inadvertently contributed to detrimental individual level outcomes.

In addition to the economic ramifications of social distancing, limiting in-person interactions also takes an emotional toll [4]. As social beings, the presence of healthy interpersonal relationships has long been viewed as essential to psychosocial functioning [5–7] given our innate “need to belong” [8]. Multiple mechanisms of maintaining social connectedness (e.g., human companionship, affection exchange, and social support) have established benefits for mental health [9–12]. In contrast, loneliness increases depression and anxiety risk [13, 14]. Given that affection deprivation and loneliness are distinct constructs [15], both could contribute to social distancing’s negative effects on mental health. Consequently it is unsurprising that empirical evidence suggests that social distancing during recent, smaller pandemics precipitated depression and anxiety [16, 17]. Initial data suggest that lockdown and distancing during COVID-19 has resulted in similar effects [4]. Luckily, social connectedness is not solely dependent on physical presence, but also develops through meaningful interactions [18]. That said, although technology enables virtual interaction (Facetime, Google Chat, Skype, Zoom) these substitutions do not enhance mood and social belonging to the extent that face-to-face interactions do [19, 20].

Unfortunately, social connectedness does not guarantee mental health. Some interaction strategies, including co-rumination and social media use may inadvertently foster risk for internalizing disorders such as depression and anxiety [21–24]. There are multiple means of seeking support and maintaining interpersonal relationships that individuals may choose to voluntarily engage in while social distancing. Because interaction strategies reflect voluntary choices, distinguishing adaptive from maladaptive strategies may be a critical means of maintaining both mental health and relationships across the course of this pandemic. The stress of social distancing in addition to navigating a pandemic may exacerbate the harmful effects of maladaptive strategies. Further, interaction strategies during a pandemic that requires social distancing may pose unique risks for fostering health anxiety. To our knowledge, health anxiety, or attributing normal bodily sensations and occurrences to perceived or suspected disease states [25] has not yet been linked to interpersonal interaction strategies.

### Social media and mental health

The absence of face-to-face contact has necessitated alternative means for social interaction. Most adults used social media daily prior to the COVID-19 pandemic, with Facebook being one of the most common platforms (69% of US adults) [26]. We expected reliance on social media to increase during quarantine since feeling socially disconnected drives social media use [27]. Much research published since the beginning of the pandemic indicates that media use has increased from pre-pandemic times [28–30]. However, there are adjustment trade-offs with social media use. Benefits can include increasing connectedness, belonging, perceived social support, self-esteem, happiness, and life satisfaction [27, 31–35]. Conversely, social media use has been linked to higher depression and anxiety symptoms [36–39] and limiting social media use can decrease loneliness and depression [40]. In at least one study, data suggest that using social media as a coping strategy to substitute for in-person interaction made people feel less (rather than more) happy [28]. Given this mixed research, it is likely that *how* social media is used may determine risk versus benefit.

There are potential benefits to be harnessed from social media to maintain psychosocial health during the COVID-19 pandemic if maladaptive aspects are avoided. Engaging in social comparison and seeking feedback on social media decreases self-esteem, body image, and increases depressive symptoms [41–43]. Conversely, interacting with friends on social media increases happiness and belonging [34], especially when avoiding social comparisons [44, 45]. Social media use can also help with COVID-19 related mood regulation [28]. On the other hand, focusing extensive time on negative media content can lead to poorer mental health [30, 46–49], including perception of disease threat and personal risk [50]. Similar effects may occur on social media platforms [51].

A growing body of work focused on media exposure during the COVID-19 pandemic indicates that there are numerous negative consequences associated with mediated coping strategies people have adopted during the pandemic [30, 48, 52, 53]. For instance, initial data suggests exposure to COVID-19 related media generally [30] and social media specifically [53, 54], is associated with negative outcomes such as acute stress, depression, and anxiety. In another study, new media use (online, text, and social media) was associated with negative psychological outcomes such as negative affect and depression whereas the same effect was not there for traditional media (television, radio, and newspapers) use [46]. One explanation for these effects is that social media outlets often afford unrestricted access to content of questionable quality and increased sensationalizing [46, 52]. Social media may also be more rife with conflicting content which has shown to be particularly harmful [30]. In fact, COVID-19 related misinformation rampant on social media has been termed an “infodemic” [52] that increases anxiety among consumers of social media. Furthermore, social media is more engaging and can therefore be more powerful with respect to psychological effects [29]. Finally, it appears that online COVID-19 information seeking is associated with negative mental health [49, 52] suggesting that COVID-19 targeted media interaction is not the same as general media utilization. In fact, over-active engagement with health related media has been labeled “cyberchondria” [48] and has been associated with health anxiety in the context of COVID-19. Therefore, engaging with social media focused on COVID-19 may outweigh any social benefits and inadvertently foster internalized distress, particularly health anxiety.

### Co-rumination

Not all interaction strategies maximize the benefits of intimate supportive relationships. Even among positive, validating relationships, seeking support via ineffective means can foster risk for internalizing disorders. Co-ruminating or engaging in extensive problem-talk with peers focused on negative emotions and dissecting the causes and potential consequences of problems [55], increases risk for depression and anxiety in adolescents and young adults [21, 22, 56, 57]. Co-rumination is a poor emotion regulation strategy that serves to maintain negative affect [58] and increase cortisol levels [59, 60]. A definitional hallmark of co-rumination that differentiates it from other supportive interactions that co-ruminative discussions focus almost entirely on problems and negative reactions. As such, interactants do not focus on problem-solving, coping, or other proactive foci and therefore co-rumination functions to exacerbate rather than resolve problems [61]. Despite inducing biological stress and emotional distress, co-rumination appears to be socially reinforced via emotional validation and increasing friendship quality [57, 62] and thus masquerades as beneficial social support [63]. Results of at least one study indicate that COVID-19 focused co-rumination may be detrimental to mental health [64]. However, those results are subject to further examination given the contradictory findings across time points. Furthermore, to date, the effects of co-ruminating on health anxiety have yet to be examined.

Since co-rumination focuses on stressful topics, and the COVID-19 pandemic is a major life stressor, co-rumination on COVID-19 or the effects of the pandemic with peers likely confers similar adjustment trade-offs. Re-hashing negative emotions, fears, and potential consequences of the pandemic with peers will likely be associated with heightened internalizing symptoms, especially health anxiety. Although unlikely, it is possible within a quarantine environment, the benefits of increasing social connectedness and validating aspects of co-rumination may be the primary outcome. Initial evidence suggests co-ruminating via text and phone is only associated with friendship quality, not anxiety [65]. Understanding the impact of co-ruminating is warranted because effects may have a meaningful impact on wellbeing and the phenomenon is not yet fully understood in this context [64].

### The present research

The current study examined whether voluntary, modifiable interaction strategies such as how people utilize social media, were associated with mental health outcomes during the initial quarantine. Adults were recruited via social media platforms to participate in a study on social distancing during quarantine. Given that the most popular social media platform (other than YouTube) is Facebook (69% of US adults) [26], we targeted recruitment efforts through Facebook to maximize sample representation. We examined frequency and focus of social media use, tendency to co-ruminate with peers on COVID-19, and mental health: current depressive symptoms, state anxiety, and current health anxiety. Given that COVID-19 posed a specific threat to public health, it was important to capture to what extent participants felt their health anxiety had been amplified during events in the initial quarantine lockdown. Since we could not anticipate the pandemic (and thus did not have pre-pandemic measures of health anxiety already collected) we asked participants to report on their health anxiety 6 months ago (Fall 2019). Retrospective report has demonstrated utility with other forms of anxiety during the initial lockdown [48]. However, the very real mood [66] and memory biases [67] inherent in retrospective reports limits the capacity to examine objective changes in health anxiety. Rather, we term this item perceived retrospective health anxiety, as we may only surmise whether participants perceived a change. Our first aim was to examine the potential maladaptive effects of common communication strategies on mental health. We hypothesized the following:

- H1: Greater tendency to co-ruminate on COVID-19 would be associated with poorer mental health including higher current: depressive symptoms, state anxiety, and health anxiety.
- H2: Greater social media use focused on COVID-19 would be associated with poorer mental health including higher current: depressive symptoms, state anxiety, and health anxiety.
- H3: Greater co-rumination on COVID-19 and social media use focused on COVID-19 would be associated with perceived increases in health anxiety.

With prior research supporting adjustment trade-offs of both co-rumination and social media (increased friendship quality and social connectedness), our second aim was to examine whether either strategy was associated with benefits during social distancing measures. We examine associations with loneliness and perceived social support but were hesitant to hypothesize direction of associations given the complex relationship between co-rumination with social support [63]. Thus, analyses probed the following research questions (RQ).

- RQ1: Examine whether greater co-rumination on COVID-19 and social media use focused on COVID-19 were associated with higher perceived social support.
- RQ2: Examine whether greater co-rumination on COVID-19 and social media use focused on COVID-19 were associated with lower loneliness.

Finally, with the COVID-19 pandemic having unprecedented economic impacts on unemployment and thus family income (known risk factors that impact mental health), our final aim was to examine the robustness of associations between communication strategies and mental health outcomes and psychosocial adjustment by covarying for demographic risk factors.

- RQ3: Does co-ruminating on COVID-19 and social media use focused on COVID-19 continue to be associated with higher depressive symptoms, state anxiety and health anxiety after controlling for the effects of demographic risk factors?
- RQ4: Does co-ruminating on COVID-19 and social media use focused on COVID-19 continue to be associated with perceived social support and loneliness after covarying for demographic risk factors?

## Methods

### Participants and procedure

Adults 18 years and older were recruited via ads on Facebook as well as announcements on Instagram to participate in a study examining their experience with social distancing. Participants were recruited through social media to maximize the likelihood of including active social media users in the sample, given the focus of the project. The ads/announcements on both platforms included a link which directed potential participants to the informed consent page in Qualtrics.com which reviewed the ethical risks and benefits of participation. Respondents who provided consent were then asked to provide demographic data and complete surveys on mental health and social media use. Since we cannot know how many individuals saw the ads on each platform, only how many interested individuals clicked on the ad and proceeded past the informed consent page, participation rate reflects an overestimate given many individuals saw the ad and chose to not participate. Of 484 visitors to ‘consent’, 345 completed the survey (71% of consenting respondents). The final page offered the option of participating in a drawing to win one of five, $25 egiftcards. Most respondents completed the survey within 7–21 min (*M* = 14 min, *SD* = 7). Participation roughly aligned with the timeline of initial quarantine measures in the continental US. Recruitment started March 23rd and ended May 13th 2020. All study materials and procedures were approved by the institutional review board at Christopher Newport University.

Demographic data for the 345 participants are displayed in Table 1. Most participants were American (91%), women (81%) who identified as white (87%). Most reported full-time (47%) or part-time (26%) employment prior to the COVID-19 pandemic shutdowns, and 33% reported currently completing college or university. Most reported being currently single (47%) married or living with a partner (48%). Annual income ranged widely ($0—750,000) with the median income $72,000/year (*M* = $91,327, *SD* = $77,254), although 16% chose not to report.

**Table 1:** Sample characteristics and bivariate associations with current mental health

Demographics	Range	Mean	SD		CESD	STAI	HAI
Age	18–73	32.22	11.95	*r*	− .23***	− .15**	− 0.10
Gender, *n* (%)							
Woman		280	81%	*t*	0.25	1.14	0.73
Man		56	16%				
Trans/Queer		9	3%				
Race,* n* (%)							
White		302	87%	*t*	− 0.87	− 0.55	− 0.98
Black		9	3%				
Asian		11	3%				
Native American or Pacific Islander		1	< 1%				
Multi-racial		14	4%				
Other		7	2%				
Student, *n* (%)		112	33%	*t*	3.04**	1.63	0.80
Marital Status							
Single		162	47%	*t*	− 3.07**	− 1.53	− 1.96*
Married		127	37%				
Living w. partner		39	11%				
Div/Sep/W		17	5%				
Living in home	0–9	2.04	1.38	*r*	.04	.01	− .08
Income (k)	0–750	91.33	77.25	*r*	− .19**	− .05	− .08
Financial Health	1–5	2.57	1.16	*r*	.32***	.34***	.15**
Employment							
Full-time		162	47%	*ρ*	− .17**	− .07	− .02
Part-time		89	26%				
Unemployed		59	17%				
Service Industry		25	7%				
Retired		8	2%				
Pay status (*n* = 301)							
Full wages		200	67%	*ρ*	− .14*	− .12*	0.03
Partial wages or PTO		26	9%				
Filed unemploymt/no wages		72	24%				

For analyses the following variables were categorized: Gender: male (0) female (1). Race: white (0) non-white (1). Student: no (0), yes (1). Marital status (0) single, (1) Married or Living with partner. Employment status: (2) full-time, (1) part-time, (0) unemployed, service industry. Pay Status: (2) full wages, (1) partial wages or POT (personal time off), (0) public assistance or no wages. Income was converted into eight, $15,000 categories starting with: $0–15,000 (0), and ending with $105,000 and above (8)

*Trans/Queer* transgender, nonbinary or genderqueer, *CESD* depressive symptoms, *STAI* state anxiety, *HAI* current health anxiety, *ULS* loneliness

***p < .001, **p ≤ .010, *p ≤ .05

#### Demographic variables and assessment of financial health

For marital status, participants were asked to identify whether they were: single, married, unmarried living with partner, divorced or separated, or widowed. As displayed in Table 2, several categories were too small to examine. To examine the impact of living with a significant other vs. alone, for analysis this variable was recoded: (0) single, (1) married or living with partner.

**Table 2:** Correlations and Descriptive Statistics of Social Regulation Predictors and Mental Health Outcomes

Variables	1	2	3	4	5	6	7	8	9	10
1 CRQ	–									
2 SM	.03	–								
3 Posts	.06	.26***	–							
4 SMCOVID	.30***	.41***	.14**	–						
5 CESD	.32***	.27***	.16**	.26***	–					
6 STAI	.34***	.21***	.10	.32***	.73***	–				
7 PR-HAI	.10	.12*	.09	.11*	.30***	.26***	–			
8 C-HAI	.25***	.08	− .02	.17**	.32***	.35***	.65***	–		
9 ULS	.19***	.19***	.12*	.11*	.58***	.43***	.18***	.20***	–	
10 SS	− .01	− .05	− .09	− .09	− .27***	− .25***	− .14**	− .06	− .45***	–
Mean	2.54	4.35	20.64	1.70	14.70	16.01	2.48	3.01	14.39	4.04
*SD*	0.76	2.93	71.31	1.87	6.58	4.28	0.68	0.48	3.92	0.75

For clarity, means and standard deviations of non-transformed variables are displayed

*CRQ* co-ruminating on covid19, *SM* time spent on social media, *SMCOVID* Time spent on social media focused on covid19, *CESD* Depressive symptoms, *STAI* current anxiety, *PR-HAI* perceived retrospective health anxiety, *C-HAI* current health anxiety. *ULS* UCLA loneliness scale, *SS* social support

***p ≤ .001, **p < .01, *p < .05

Employment status was assessed by asking participants to identify their current situation among 5 categories: full-time salaried employee; part-time employee; unemployed (including students); service industry employment (based on hourly wages/commission); retired. Based on representation across categories, Employment status was recoded into an ordinal variable for analyses: (2) full-time, (1) part-time, (0) unemployed, service industry.

We also examined the impact of current pay given that many individuals had been furloughed or were attempting to receive public benefits. Based on participants’ responses we coded Pay Status into an ordinal variable for analyses: (2) full wages, (1) partial wages or POT (personal time off), (0) public assistance or no wages.

Participants were asked to enter what their household annual income was, because the income skew was so extreme (ranged between $0—750,000), Income was converted into an interval variable with nine categories with a $15,000 range starting with: $0–15,000 (0) and ending with $105,000 and above (8).

Financial health was assessed via two questions: ‘*How concerned are you about your ability to: purchase food, supplies, medicine for the next several weeks or months?’* and ‘*continue making payments on your mortgage, credit cards, utilities for the next several weeks or months*?’ Responses were recorded via a 5-point scale ranging from 1 (not at all concerned) to 5 (extremely concerned). Average financial health was computed, α = 0.81.

#### Self-report measures of mental health and loneliness

Depressive symptoms were assessed with a 10-item, shortened version of the Center of Epidemiologic Studies Depression scale CES-D [68], which was designed to assess depression symptoms in the general population [69], α = 0.86. The CES-D assesses depressive symptoms, *‘I was bothered by things that usually don’t bother me’,* in the past week on a 4-point likert-type scale ranging from ‘rarely or none of the time’ (0) to ‘most or all of the time’ (3). Two items were reverse scored, then all items were summed with higher scores indicating higher depressive symptoms.

State anxiety was assessed via the state anxiety subscale of the 6-item version of the State Trait Anxiety Inventory [70]. Items ask participants the rate their current status, *‘I feel worried*’ on a 4-point Likert scale ranging from *‘not at all’*(1) to *‘very much’* (4). Three items are reverse-scored, then items are summed so that higher scores indicate higher current anxiety, (α = 0.87).

Healthy Anxiety was measured via the 5-item Fear of Illness subscale of the Health Anxiety Inventory (HAI) [71]. Items assess how frequently participants’ worry about their health on a 4-point Likert-type scale ranging from 1 to 4. For example: ‘*As a rule I am not afraid that I have a serious illness’* (1); ‘*I am sometimes afraid that I have a serious illness’* (2); ‘*I am often afraid that I have a serious illness’* (3); and *‘I am always afraid that I have a serious illness’* (4). The average is taken across all items, with higher means indicating higher health anxiety. As has been done in other research on COVID-19 health anxiety [48], to assess changes in health anxiety, participants completed the HAI twice: over the past six months (perceived retrospective or PR-HAI: α = 0.78), and in the past week (Current-HAI: α = 0.83).

Loneliness was measured via the 6-item short-form UCLA Loneliness Scale (ULS: α = 0.83) [72]. Items assess current loneliness, ‘*I lack companionship’* on a 4-point scale ranging from ‘never’ (1) to ‘always’ (4). Items are summed with higher scores indicating higher loneliness.

#### Psychosocial assessment

*Social support* was assessed via the 6-item, brief form of the Perceived Social Support Questionnaire (F-SozU: α = 0.77) [73]. Items assess the extent to which participants perceive support available, *‘I know a very close person whose help I can always count on’*. Answers ranged from ‘*not at all true*’ (1) to *‘very true*’ (5) and were averaged such that higher means indicate higher perceived social support.

Social distancing was assessed by asking participants, ‘*I have limited my contact with friends, loved ones, or acquaintances’*. Answers ranged from 1 (strongly agree) to 5 (strongly disagree), anyone answering 1 or 2 was considered to be actively social distancing. We also asked how many individuals currently live with them (SocialHome).

Social Interaction was assessed by asking participants to estimate how much they relied on the following methods to interact with friends and loved ones: (a) Social media (Facebook/ Twitter/ Instagram), (b) texting, (c) talking on the phone or ‘facetiming’ (d) in person. The percentages had to add up to 100%. Participants reported on their methods ‘pre-coronavirus’ and ‘in the past week’.

#### Self-report measures of social media use and co-rumination on COVID-19

Participants were asked how many hours they spend each day on social media (TimeSM) including time reading/viewing others’ profiles and content, *M* = 4.35 h, *SD* = 2.93. They were then asked to estimate what percentage of that time was spent focused on COVID-19 content, answers ranged from 0 to 100% on time online. To calculate the amount of time spent on social media focused on COVID-19 (SMCOVID), TimeSM was multiplied by percentage of time focused on COVID-19. For example, if a participant reported spending 3 h a day on social media, and 50% of that time was focused on COVID-19, SMCOVID = 3 × 0.50, or 1.50.

The 9-item short-form of the Co-rumination Questionnaire CRQ [56], was adapted to assess co-rumination focused on COVID-19. The CRQ asks about problem-focused talk. Items were amended, with ‘problems’ replaced by ‘coronavirus’. For example, ‘*If one of us has been thinking about a problem, we will talk about the problem rather than talking about something else or doing something else’* was changed to: ‘*If one of us has been thinking about coronavirus, we will talk about coronavirus rather than talking about something else or doing something else’* Internal reliability in the current sample was excellent, α = 0.85.

#### Ongoing impact of the COVID-19 pandemic

Acknowledging that the impact of COVID-19 has evolved overtime we also created a variable detailing rates of new COVID-19 cases in the US on the date of participation [74].

## Results

### Data preparation and preliminary analyses

Several variables were skewed; square root (CRQ), logarithmic (PR-HAI, Current-HAI; Social support; TimeSM; PostsSM; SMCOVID) and inverse transformations (daily COVID-19 cases) were applied to meet assumptions of normality. There was up to 1.2% of missing data (or *n* = 4) among primary variables (communication strategies and mental health outcomes). Missing cases were excluded pairwise across analyses. Prior to analyses we first considered whether the timing of participation impacted mental health outcomes. Rates of daily COVID-19 cases were not significantly associated with depressive symptoms, state anxiety or current health anxiety lowest *p* = 0.156. Thus, bivariate associations between demographic variables and mental health outcome measures are presented in Table 1. Pearson’s *r* was used to examine the correlation between continuous variables, Spearman’s *ρ* for ordinal (employment and pay status), and categorical variables (e.g., gender and race) were tested via independent samples *t* tests.

**Fig. 1:**
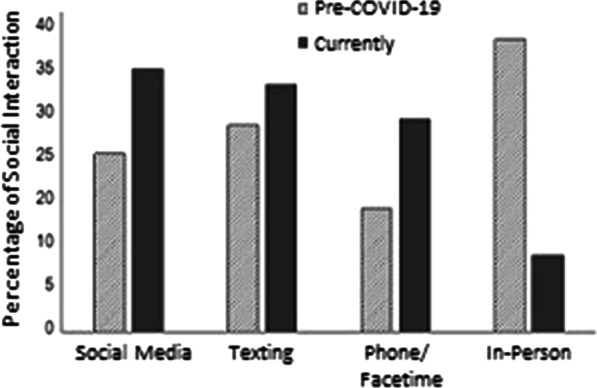
Social interaction methods changed during quarantine

Most participants (92%) reported actively social distancing. We first examined relative changes in social interaction mediums via paired samples *t* tests. Social media use significantly increased compared to pre pandemic levels, *t*(344) = 12.00, *p* < 0.001, as did texting, *t*(344) = 6.67, *p* < 0.001, and phone use, *t*(344) = 13.30, *p* < 0.001. In contrast, in-person interactions decreased, *t*(344) = -25.02, *p* < 0.001. Results are displayed in Fig. 1.

### Testing primary hypotheses

#### H1

Co-ruminating on COVID-19 would be associated with poorer mental health.

Bivariate associations for H1 and H2 are displayed in Table 2. Consistent with the hypothesis, greater tendency to co-ruminate on COVID-19 with peers was associated with higher current depressive symptoms, state anxiety, and health anxiety.

#### H2

Social media use focused on COVID-19 would be associated with poorer mental health.

Consistent with the hypothesis, spending more time on social media focused on COVID-19 was associated with higher current depressive symptoms, state anxiety, and health anxiety. In contrast, general indices of social media use were less consistent predictors of mental health. Greater social media interaction (posts) was only associated with higher depressive symptoms. Overall time spent on social media was linked to higher depressive symptoms and state anxiety.

#### H3

Co-ruminating on COVID-19 and social media use focused on COVID-19 would be associated with perceived increases in health anxiety.

First, we examined whether participants perceived a change in their health anxiety over the past 6 months. A paired samples *t*-test revealed that across the full sample, participants perceived their current health anxiety to be significantly higher than six months prior, *t*(325) = 9.61, Cohen’s *d* = 2.43. To determine whether communication strategies corresponded with differences in perceived changes in health anxiety, two hierarchical regressions were run covarying for (controlling for the effects of) perceived retrospective health anxiety. PR-HAI was entered on step 1 and then the primary predictor was entered on step 2. Aligning with hypothesis, higher co-ruminative tendency was associated with larger perceived increases in health anxiety, *β* = 0.18, *t*(342) = 4.54, *p* < 0.001, *sr* = 0.24. Similarly, greater time spent on social media focused on COVID-19 was also associated with larger perceived increases in health anxiety, *β* = 0.10, *t*(342) = 2.35, *p* = 0.019, *sr* = 0.13.

### Secondary aim: examine interpersonal benefits of maladaptive communication strategies during social distancing

#### RQ1

Are co-ruminating on COVID-19 and social media use focused on COVID-19 associated with higher perceived social support?

As displayed in Table 2, neither communication strategy correlated with perceived social support.

#### RQ2

Are co-ruminating on COVID-19 and social media use focused on COVID-19 associated with lower loneliness?

Both co-ruminating on COVID-19 and social media use focused on COVID-19 were correlated with higher loneliness.

### Third aim: examine robustness of associations

#### RQ3

Does co-ruminating on COVID-19 and social media use focused on COVID-19 continue to be associated with higher current depressive symptoms, state anxiety and health anxiety after controlling for the effects of demographic risk factors?

Specificity tests were conducted via three hierarchical regressions to identify if communication strategies accounted for unique variance in current depressive symptoms, state anxiety and health anxiety when controlling for the effects of significant demographic variables. Demographic covariates were entered on step 1 and then the primary predictors were entered on step 2. For example, student status was entered as a significant covariate of depressive symptoms but not state anxiety or health anxiety. This approach maximized model parsimony for each of the three hierarchical regressions (on depressive symptoms, state anxiety and health anxiety). The variance inflation factors (VIF) for all three models were under 2.5, indicating low risk of multicollinearity. Results are displayed in Table 3. Both co-ruminating on COVID-19 and greater time on social media focused on COVID-19 continued to account for unique variance in current depressive symptoms, state anxiety and health anxiety. Demographic factors including lower age and poorer financial health also continued to correlate with poorer mental health outcomes.

**Table 3:** Specificity analyses: hierarchical regressions predicting current mental health

Significant bivariate Predictor	CESD *β*	STAI *β*	HAI *β*	ULS *β*
Age	− .17*	− .12*	–	− .18*
Student	− .02	–	–	− .07
Marital status	− .02	–	.16**	.01
Income	− .06	–	–	.03
Financial health	.16*	.19***	.11*	.02
Employment	.03	–	–	.17*
Pay Status	− .08	–	–	− .13
Co-rumination	.20**	.22***	.20***	.08
Social media	.14	.11	–	.10
Social media, COVID-19	.14*	.20**	.11*	.00
Social media posts	.19*	–	–	.18*
Social support	− .21***	− .17**	–	− .44***
*R*^2^ change	0.32***	0.30***	0.11***	0.32***
Highest VIF	2.18	1.56	1.12	1.79

*CESD* depressive symptoms, *STAI* anxiety symptoms, *HAI* current health anxiety, *ULS* loneliness, *VIF* Variance inflation factor

***p ≤ .001, **p ≤ .010, *p ≤ .05

Finally, we also examined the robustness of effects on perceived changes in health anxiety by running two hierarchical regressions while covarying for demographic factors significantly correlated with current health anxiety (marital status and financial health) as well as perceived retrospective health anxiety. Both communication strategies continued to predict greater perceived increases in health anxiety: social media focused on COVID-19, *β* = 0.10, *t* (340) = 2.50, *p* = 0.013, *sr* = 0.14; and co-ruminating on COVID-19: *β* = 0.18, *t*(340) = 4.40, *p* < 0.001, *sr* = 0.24.

#### RQ4

Is Co-ruminating on COVID-19 and social media use focused on COVID-19 still associated with perceived social support and loneliness after covarying for demographic risk factors?

An additional hierarchical regression was run to examine specific effects with loneliness. As displayed in Table 3, neither communication strategy was significantly correlated with loneliness after controlling for the effects of demographic risk factors.

## Discussion

The current study examined the association between mental health and the social interaction strategies individuals adopted during the initial wave of the COVID-19 pandemic in Spring 2020. Most participants were actively social distancing: face-to-face interactions had drastically decreased while reliance on social media, phone-use, and virtual interactions increased. Two social connection strategies, co-ruminating on COVID-19 during interactions with peers and spending time on social media focused on COVID-19 were associated with poorer current mental health outcomes as well as changes in perceived health anxiety. Further, neither strategy was associated with social benefits, indeed both were associated with greater loneliness. The current pattern of results extends prior research indicating that neither strategy exhibits adjustment tradeoffs in the context of quarantine when face-to-face interaction is limited.

Finding that co-ruminating on COVID-19 was associated with higher depressive symptoms, state anxiety, and health anxiety during quarantine extends prior research, indicating that even in the context of social distancing, co-rumination is still associated with heightened risk for internalizing symptoms. Pre-pandemic research supports that co-ruminating with peers increases risk for developing depression and anxiety [21, 22, 56, 57], and is likely reinforced through social benefits such as increasing relationship quality [57]. Given the constraints on face-to-face interaction during quarantine, one possibility was that in this strained context, co-ruminating could post less affective risks while still meeting social connection needs. Thus, it is striking that co-ruminating on COVID-19 in the context of social distancing was still associated with heightened depression and anxiety symptoms while failing to be linked with the social benefits. Participants who tended to engage in greater co-rumination did *not* report less loneliness or greater social support, instead they reported higher levels of loneliness. A potential explanation for this pattern is that co-ruminating tends to increase stress response [59], and exacerbate problems [56, 61]. Thus, if participants’ co-ruminative exchanges focused on the loneliness that they were experiencing, it stands to reason that in the context of social distancing during COVID-19, these interactions could magnify, rather than decrease, feelings of loneliness. It is worth emphasizing though that causal effects between interaction strategies and mental health outcomes cannot be inferred given the cross-sectional study design. However, given the established role of co-rumination as a risk factor in non-pandemic times: the current pattern of results are worrisome, and suggest the co-rumination confers neither affective nor social benefits in the context of social distancing.

Similarly, social media use focused on COVID-19 was also associated with higher depressive symptoms, state anxiety, and health anxiety. This pattern aligns with prior research indicating that *how* social media is used is a stronger predictor of mental health than general time spent online [41–43, 46]. Finding that COVID-19 focused social media content (not general social media use) accounted for unique variance in each mental health outcome even after covarying for other social media factors and demographic variables extends research linking social media content with heightened depression, and health anxiety risk [46, 50, 54]. It is noteworthy though, social media use was not associated with *lower* loneliness, and surprisingly, greater interaction (posts) on social media was associated with higher depressive symptoms and loneliness. While our data do not allow us to examine the specific content of participants’ posts, it would stand to reason that much like with co-rumination, posting or reading about feelings of loneliness and isolation related to COVID-19 may function to exacerbate those feelings, explaining this unexpected association. However, this possibility requires longitudinal data to substantiate this proposed direction of effect. Taken together, results indicate that utilizing social media to engage with COVID-19-related content was associated with neither social nor affective benefits.

Surprisingly, there was no association between any of the interaction strategies and perceived social support. These data suggest that it is possible that in the context a COVID-19 related social isolation, co-rumination does not confer benefits of feeling more supported in the way that it does under other circumstances [61]. Similarly, it is plausible that within the context of an ongoing pandemic, social media engagement ceases to feel supportive. In sum, these findings suggest that the context within which these behaviors occur, and the topical foci of the interactions themselves, may function to suppress the extent to which they feel supportive. Therefore, this project helps clarify the conflicting findings on the adjustment tradeoffs inherent in these behaviors, although future research should further examine this possibility more directly.

A strength of the current study was capturing individuals’ coping responses and mental health status during the initial wave of the COVID-19 pandemic. A limitation is that we did not have pre-pandemic data on their mental health. Participants reported their perceived retrospective health anxiety so that we could assess whether their current social connection strategies were associated with perceived changes in distress. Everyone perceived an increase in their health anxiety over the past six months, but we found that participants who tended to co-ruminate on COVID-19 and spend time on social media focused on COVID-19 perceived their health anxiety as amplified to a greater extent than other participants. This pattern extends prior research [64], indicating that even in the context of social distancing, these strategies are still associated with maladaptive effects on health anxiety. Given the multiple limitations on retrospective reports we cannot be confident that participants experienced an increase in health anxiety. However, the perceived amplification of anxiety does align with past research showing that epidemic and pandemic events tend to increase health anxiety (especially for those already prone to health anxiety as a trait) [48]. Furthermore, the fact that participants perceived an increase in health anxiety is meaningful in and of itself given that such perceived anxiety increases result in both behavioral and psychological changes such as increased stockpiling behaviors, cyberchondria, and social withdrawal [48, 75].

An important aspect of the current results is that co-rumination and social media focused on COVID-19 were associated with depression and anxiety even after accounting for relevant demographic variables, which is a novel contribution to the literature. Risk factors such as employment status and financial health, not surprisingly, also accounted for variance in mental health outcomes. Surprisingly, the number of individuals living in the home was not associated with mental health outcomes. Thus, regardless of whether participants continued to have face-to-face interactions in the home, how they chose to mindfully engage and connect with their social networks accounted for unique variance in their mental health.

Although the current study was cross-sectional, the pattern of results linking voluntary social connection strategies with multiple poorer health outcomes is noteworthy. Given the potential direction of effect, the results may have important implications for preserving relationships and mental health while social distancing and add to the growing body of work on COVID-19 and mental health. Results indicate that it may be detrimental to maintain social connection via co-ruminating with friends on COVID-19 or focusing on COVID-19 on social media. If COVID-19 is quantified as a ‘problem’ with no immediate solution, then it makes sense why exerting extensive energy discussing thoughts and fears (co-ruminating) or reading/posting (ruminating) on social media content magnifies health anxiety and is associated with higher depression and state anxiety. Co-rumination masquerades as a functional and prosocial behavior, and we recognize the need for validation of fears and concerns. One compromise is to discuss COVID-19 concerns, but to be mindful to not let supportive interactions devolve into co-rumination by limiting COVID-focused discussion (5–10 min) and redirecting interaction outside that appointed time to other meaningful pursuits and interests. Savoring memories of positive events, small daily enjoyments, or planning upcoming activities with loved ones amplifies and maintains positive affect [76, 77], even when fewer positive events are occurring [78]. Similarly, the affective and social benefits of social media use may be harnessed by using social media primarily as a mechanism to interact with peers rather than to keep up with national news, politics etc., (e.g., perhaps ‘snooze’ the news or friends who post frequently about COVID-19 for the duration of the pandemic). For some demographics (such as with older or younger individuals), social media literacy training may be an effective strategy for helping people learn to maximize the beneficial potential of social media, while minimizing the negative effects [52]. Being strategic about social media use and being mindful of avoiding co-ruminative interactions are behaviors within one’s control and can have an especially profound effect on health anxiety, which is particularly salient in a pandemic setting. We wish to distinguish social media use from general time spent online. Research supports that digital health information seeking is associated with the adoption of preventative behaviors [79, 80]. Thus, there is genuine value of harnessing the powers of the internet to seek high quality health-related news when done in moderation. The pattern of the current results suggests that the benefits of connecting with loved ones through social media may be maximized by mindful engagement (and focusing on relationships not stress-inducing news).

### Limitations

The current study benefited from assessing interpersonal strategies during the first few weeks that social distancing measures were put in place in the continental US during the COVID-19 pandemic. That said, several limitations should be noted. We recruited through social media, requiring access to a computer or smart-device for participation. Thus, our sample aligns with typical survey recruitment, over-representing women and affluent individuals, who have historically been more likely to participate [81]. However, men and women tend to have similar social media usage rates [82], and given our focus on identifying modifiable strategies, there is value in showing an effect on well-being for those who engage with social media. Although future research on the effects of men’s social connection strategies is needed, a potential strength of over-representing women in this sample is that the pandemic has disparately impacted women. Women have left the workforce at greater rates than men due to disparate childcare-burdens at home [83], and women have also carried greater emotional burdens to protect the family since men have been more likely to adopt anti-masking policies [84]. Further, women have experienced greater vulnerability due to domestic violence and the heightened isolation of social distancing [85–87]. Women also tend to rely on co-rumination more than men in their close relationships. Taken together, understanding the link between social connection strategies and mental health outcomes among women as the primary source of domestic and care-labor is warranted. However, given economically strained individuals experienced unique threats during the pandemic [88], the current pattern of results likely will not generalize to low-income individuals with limited access to digital platforms fighting more desperate threats (daily access to clean water, food, shelter etc.). Further research is needed to clarify how/whether social connection strategies have affected low-income individuals, men, and non-binary individuals’ mental health.

Despite directly reaching out to social platforms for persons of color (POC), participation of POC was low. Racial and ethnic minorities have experienced profoundly disparate impacts during the COVID-19 pandemic for a multitude of reason (workforce layoffs, limited access to protective gear and medical care and discrimination from treatment providers) [89]. Never mind the additional burdens of advocating in social justice causes such as the Black Lives Matter movement [90] and drastic risk in hate crimes and discrimination against Asian/Asian American individuals [91]. Extensive research is needed to clarify how to harness the benefits of social support for these marginalized groups and to investigate whether the associations found here also hold for persons of color.

There are also methodological concerns worth considering. The data are cross-sectional and may be subject to recall bias. Additionally, we did not specifically assess the mechanism by which co-ruminating on COVID-19 with peers occurred. Prior research has largely assessed face-to-face co-rumination, and evidence is mixed regarding the risks and benefits of co-ruminating via different mediums [65, 92], which is important to clarify given the current need for social distancing. Regarding social media use, we did not distinguish passive (reading/view peers’ profiles/posts) versus time actively interacting with peers (posting, messaging). Given that prior research has found interaction online with peers to be adaptive, differentiating utilizing of social media time may be an important focus of future research to disentangle.

## Conclusions

The current results highlight the value of genuine human connection during times of physical isolation and suggest that at least for some populations, some voluntary interaction strategies are associated with heightened internalizing symptoms in the context of initial quarantine. Although cross-sectional in nature, results indicate that in this sample of mostly female, Caucasian, social media users, co-ruminating on COVID-19 and focusing on COVID-19 on social media was associated with perceived increases in health anxiety, higher current depressive symptoms, state anxiety and health anxiety and higher loneliness. Given prior research that co-rumination and perseverating on topics of worry amplify and maintain anxiety and depression, results have implications for maintaining psychosocial health during times when opportunities to engage in face-to-face interaction are limited. Future research is needed to examine whether these effects hold in other populations and to clarify interpersonal strategies that confer strongest adaptive effects for increasing social connectedness, friendship quality, and maintaining positive affect.

## Data Availability

The data that support the findings of this study are not publicly available due to ongoing analyses but are available from the corresponding author, L. Stone, upon reasonable request.
